# Supplementary material for: Two pathways regulate cortical granule translocation to prevent polyspermy in mouse oocytes
Source: Nat Commun. 2016 Dec 19;7:13726. doi: 10.1038/ncomms13726 (PMC5187413; doi:10.1038/ncomms13726)
Supplement: Supplementary Information — Supplementary Figures 1-6, Supplementary References [file ncomms13726-s1.pdf]

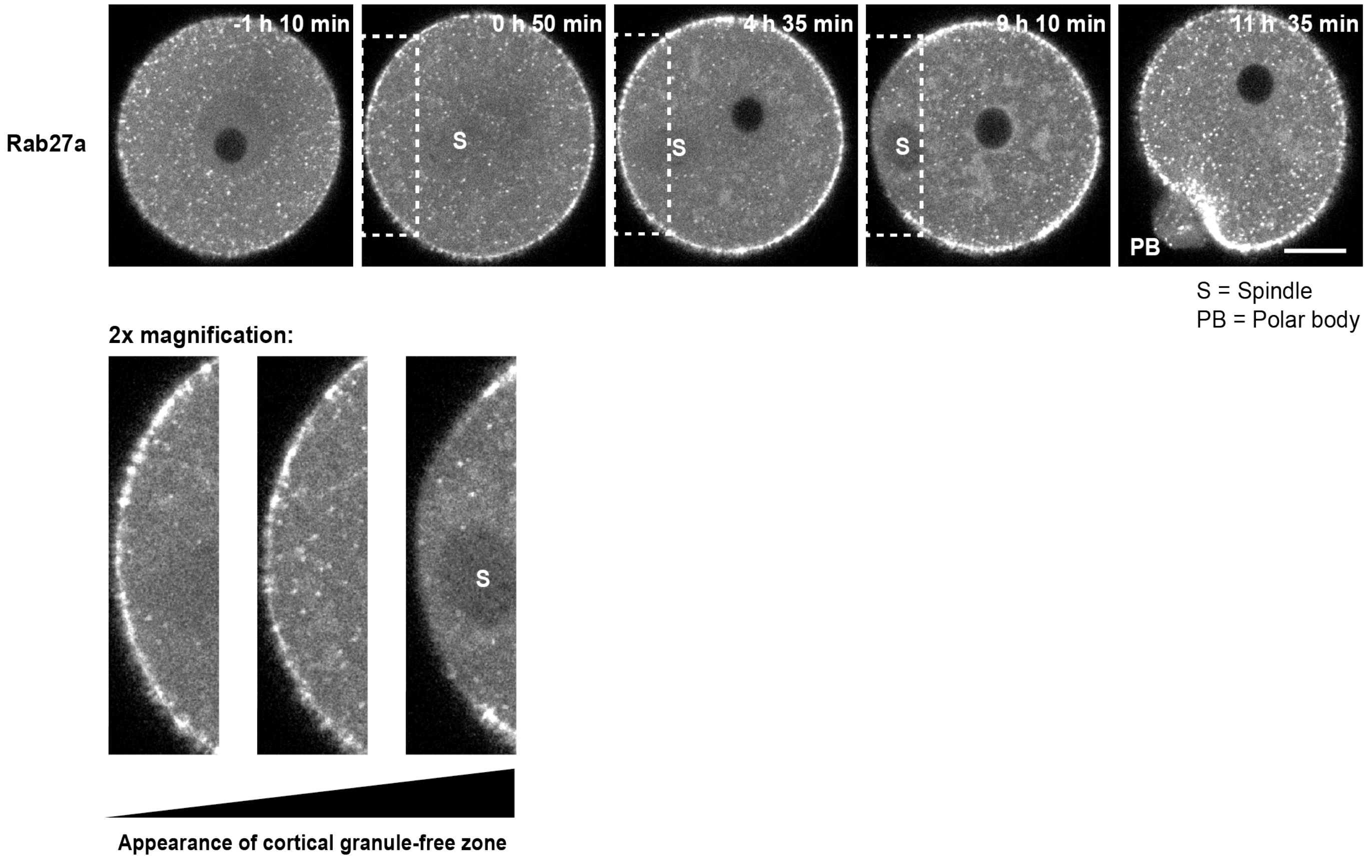

Supplementary Figure 1. Rab27a puncta follow the same characteristic patterns as cortical granules during meiotic maturation. Still images of a live oocyte expressing GFP-Rab27a during meiotic maturation. Meiotic spindle and polar body are indicated. Note the appearance of a Rab27a-free zone at the cortex adjacent to the meiotic spindle, similar to the previous described cortical granule-free zone[1] (magnification). Scale bar, 20 µm.

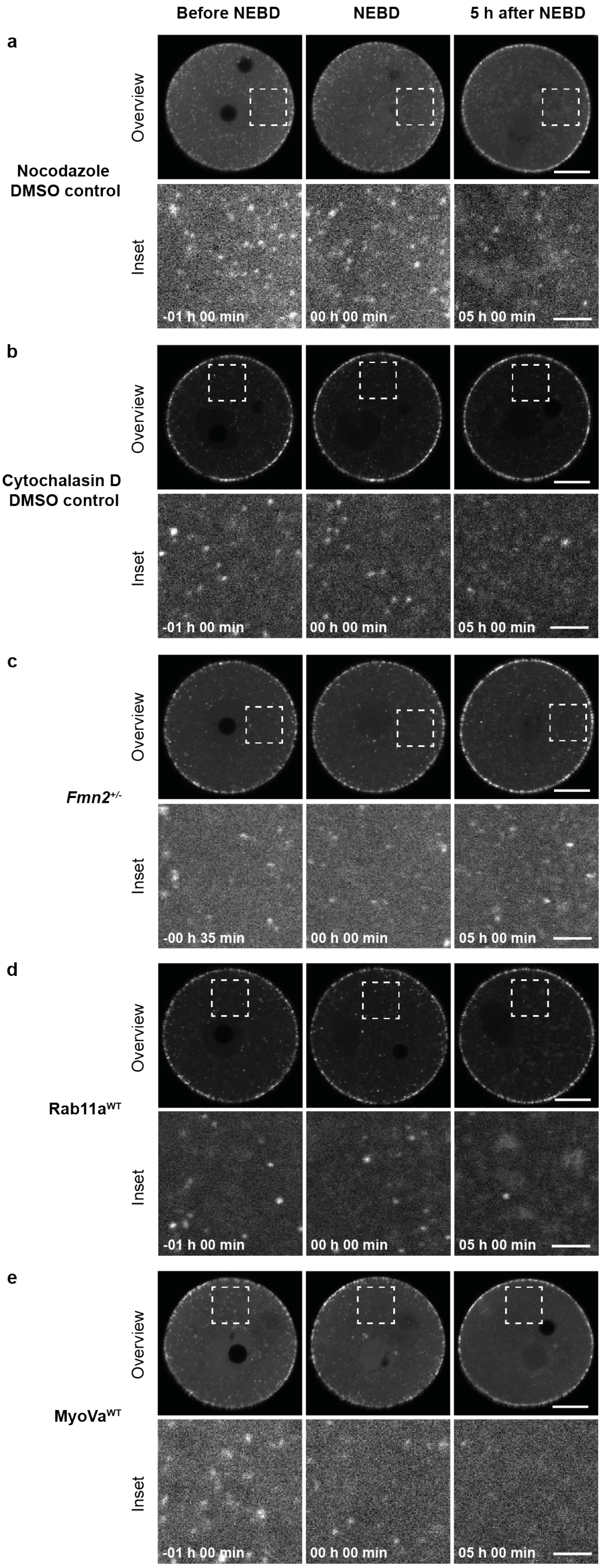
Before NEBD
NEBD
5 h after NEBD
a
Nocodazole DMSO control
Overview
Inset
-01 h 00 min
00 h 00 min
05 h 00 min
b
Cytochalasin D DMSO control
Overview
Inset
-01 h 00 min
00 h 00 min
05 h 00 min
c
Fmn2+/-
Overview
Inset
-00 h 35 min
00 h 00 min
05 h 00 min
d
Rab11aWT
Overview
Inset
-01 h 00 min
00 h 00 min
05 h 00 min
e
MyoVaWT
Overview
Inset
-01 h 00 min
00 h 00 min
05 h 00 min

Supplementary Figure 2. Cortical granule translocation in control oocytes. **(a-e)** Representative example still images (maximum intensity projections of confocal Z sections) of the control conditions for the experiments presented in Figure 3a-f, Figure 4h,i and Figure 5a,b. Scale bars, 20 µm (overview) and 5 µm (enlarged).

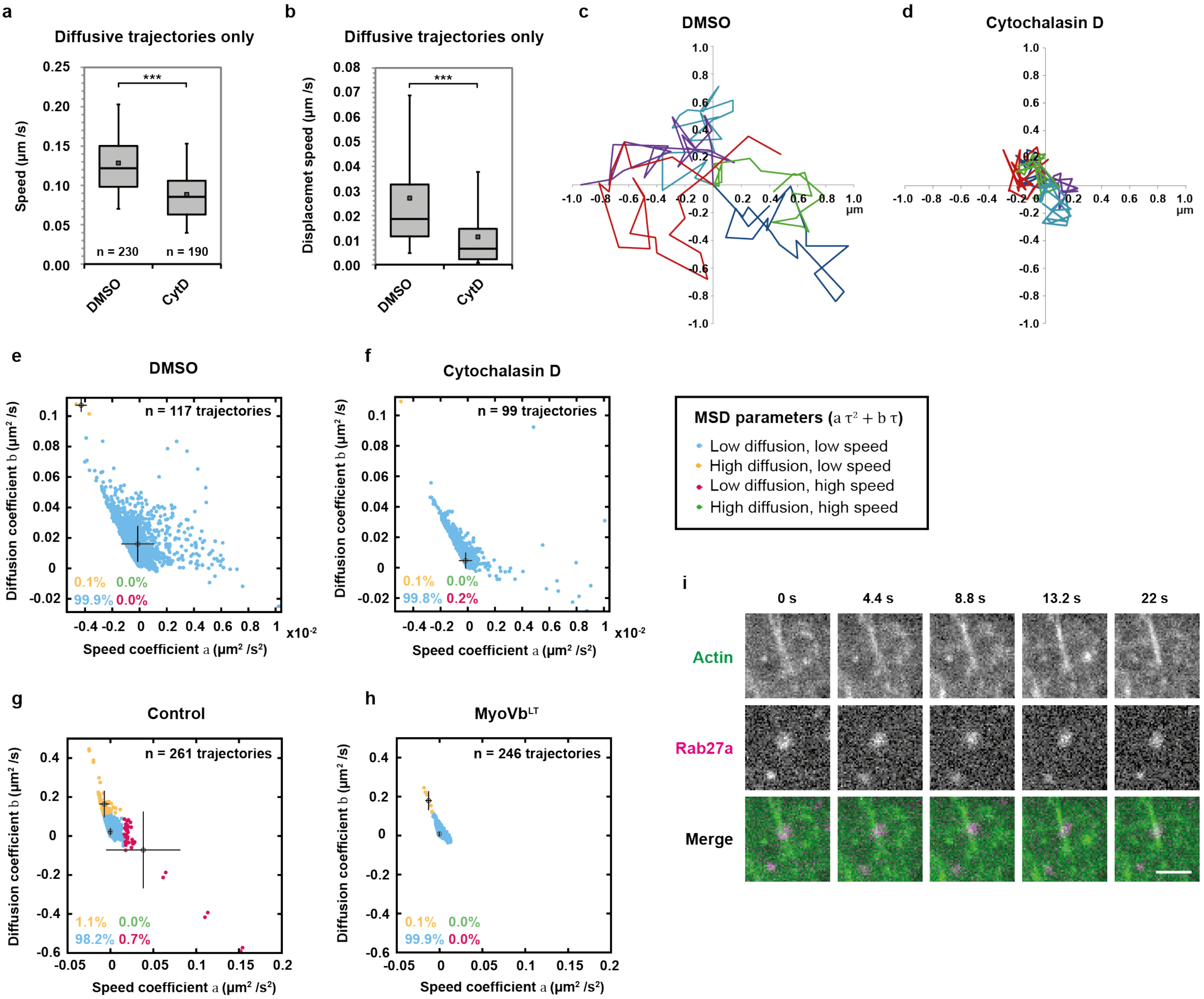

Supplementary Figure 3. Cortical granules display anomalous diffusion driven by the dynamics of the actin cytoskeleton. (**a**-**b**) Particle speed (**a**) and particle displacement speed (**b**) of diffusive cortical granules trajectories in oocytes treated with DMSO or cytochalasin D. Numbers of trajectories are indicated in **a** (Student's *t* test). Tukey box plots in **a** and **b** show the median (line), mean (small square), interquartile range, and the 5th and 95th (whiskers). (**c**-**d**) 2D projections of 5 representative example Rab27a

trajectories of diffusive motion from oocytes treated with DMSO (**c**) or cytochalasin D (**d**) are shown. All trajectories are 60 s in duration. (**e-h**) Mean squared displacement analysis of steps along diffusive Rab27a trajectories in oocytes treated with DMSO (**e**), cytochalasin D (**f**), or expressing $MyoVb^{LT}$ (**h**) or control (**g**). Black points and crosses indicate the mean ± S.D. of each category. (**i**) Still images of a cortical granule displaying diffusive motion whilst associated to an actin filament (**i**). Scale bar, 2 µm. Significance levels: *** $p < 0.001$, from 3 experiments.

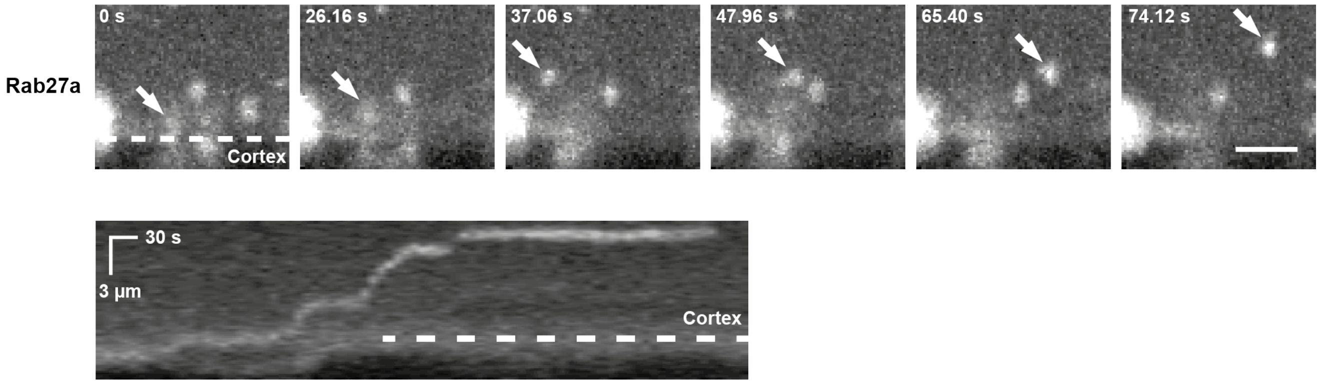

Supplementary Figure 4. Expression of the myosin Va tail domain causes the retrograde movement of cortical granules from the plasma membrane. Still images of an example cortical granule (indicated by arrows) moving in retrograde fashion from the cortex with corresponding kymograph. Scale bar, 3 µm.

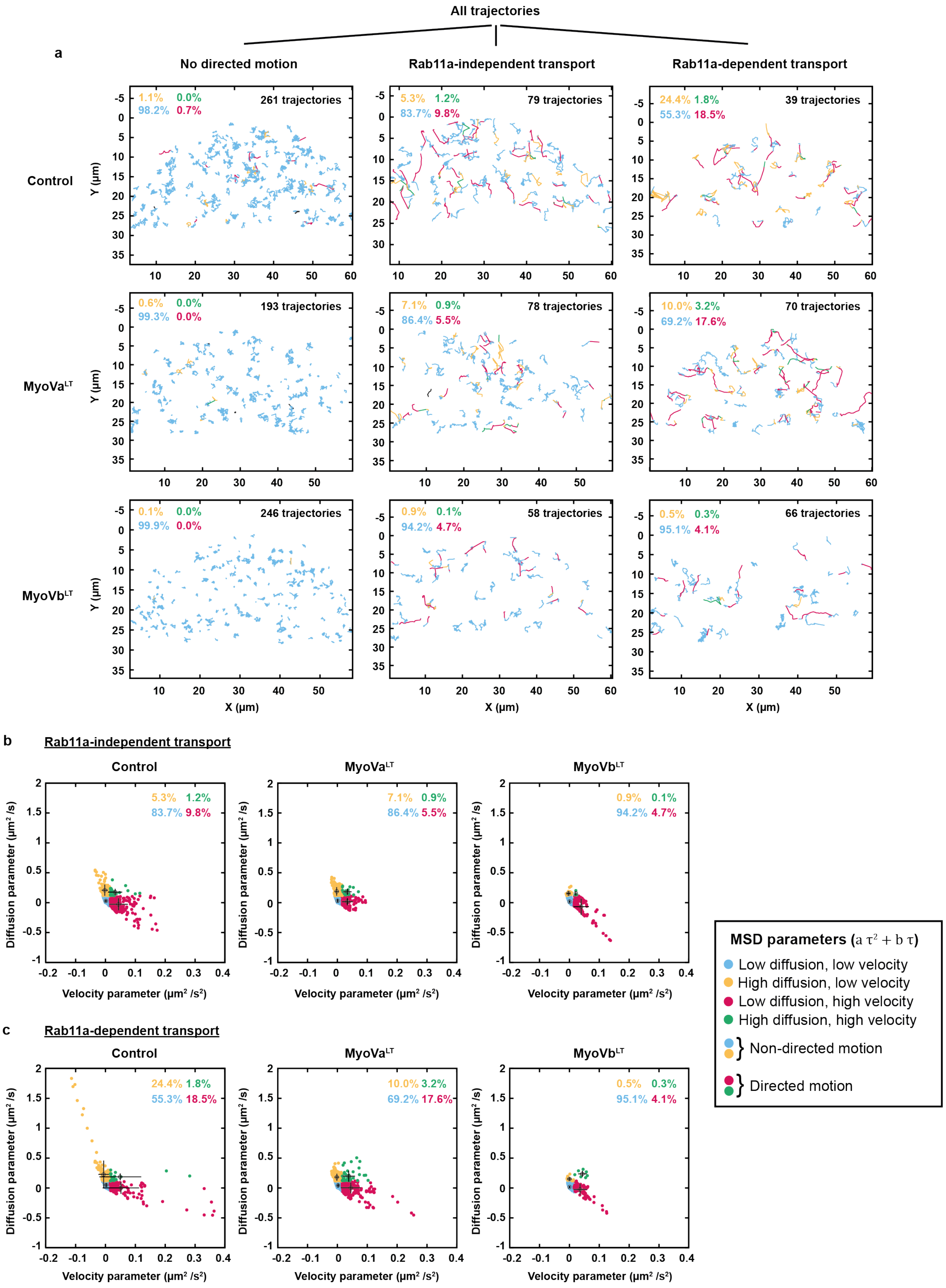

All trajectories
a
No directed motion
Rab11a-independent transport
Rab11a-dependent transport
Control
MyoVaLT
MyoVbLT
Y (µm)
X (µm)
1.1% 0.0% 98.2% 0.7% 261 trajectories
5.3% 1.2% 83.7% 9.8% 79 trajectories
24.4% 1.8% 55.3% 18.5% 39 trajectories
0.6% 0.0% 99.3% 0.0% 193 trajectories
7.1% 0.9% 86.4% 5.5% 78 trajectories
10.0% 3.2% 69.2% 17.6% 70 trajectories
0.1% 0.0% 99.9% 0.0% 246 trajectories
0.9% 0.1% 94.2% 4.7% 58 trajectories
0.5% 0.3% 95.1% 4.1% 66 trajectories
b
Rab11a-independent transport
c
Rab11a-dependent transport
Diffusion parameter (µm² /s)
Velocity parameter (µm² /s²)
MSD parameters (a τ² + b τ)
Low diffusion, low velocity
High diffusion, low velocity
Low diffusion, high velocity
High diffusion, high velocity
Non-directed motion
Directed motion

Supplementary Figure 5. Full results from **Figure 5**. (**a**) Combined Rab27a trajectories that contained Rab11a-dependent transport (hitchhiking), Rab11a-independent transport, or no active motion in oocytes expressing $MyoVa^{LT}$, $MyoVb^{LT}$, or control, after local MSD analysis. The different states of each trajectory are colour-coded as indicated in the legend, and according to the MSD results presented in **b**. Proportion of each state is indicated (%). (**b-c**) Local mean squared displacement analysis of steps along Rab27a trajectories containing either Rab11a-dependent transport (**b**), or Rab11a-independent transport (**c**) in control oocytes or oocytes expressing either $MyoVa^{LT}$ or $MyoVb^{LT}$. Thresholds separating the 4 categories of particle motion are explained in Methods. Black points and crosses indicate the mean ± S.D. of each category. Percentage of total steps are indicated for each category.

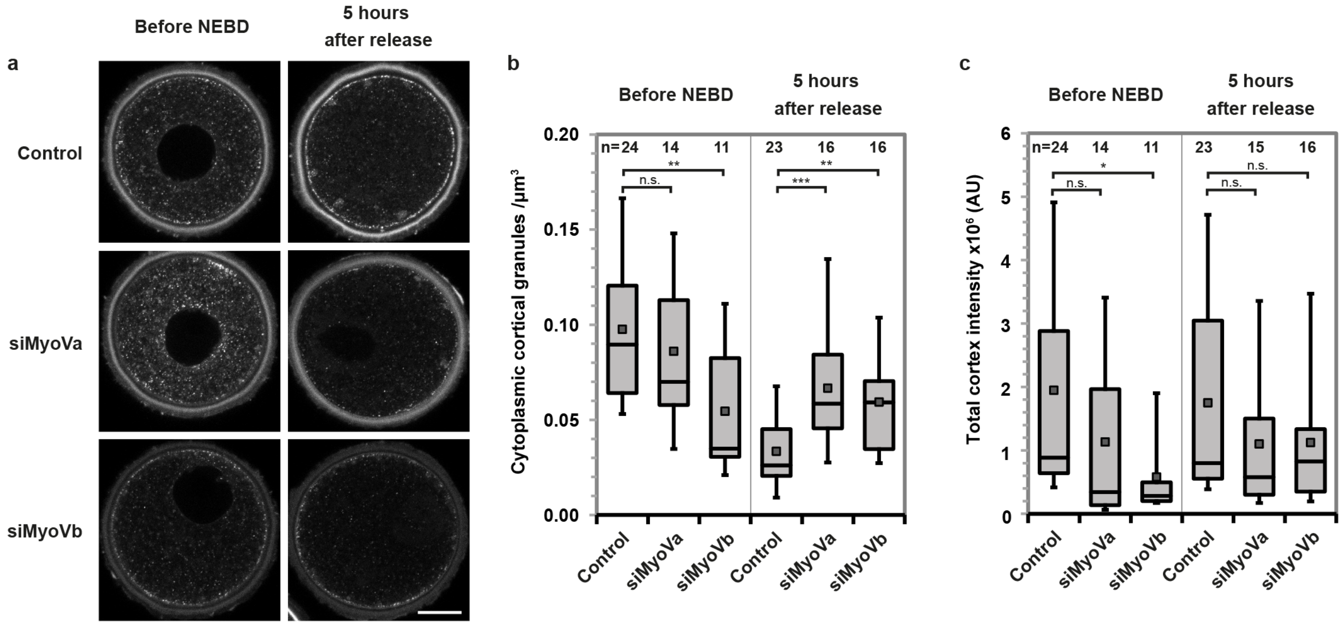

Supplementary figure 6. Depletion of Myosin Va or Myosin Vb by siRNA inhibits cortical granule translocation. (**a**) Representative example still images (maximum intensity projections of confocal Z sections) of control oocytes or Myosin Va- or Myosin Vb-depleted oocytes stained with lens culinaris agglutinin at GV stage or 5 hours after prophase release. Scale bar, 20 µm. (**b**) Quantification of cortical granules in the cell centre for the conditions in **a**. (**c**) Quantification of cortical granules at the cell cortex for the conditions in **a.** Tukey box plots in **b** and **c** show the median (line), mean (small square), interquartile range, and the 5th and 95th (whiskers). Significance levels: n.s. non-significant, * $p < 0.05$, ** $p < 0.01$, *** $p < 0.001$, using Kruskal–Wallis' ANOVA test from 3 experiments.

Supplementary references

1. Ducibella, T., Duffy, P., Reindollar, R. & Su, B. Changes in the distribution of mouse oocyte cortical granules and ability to undergo the cortical reaction during gonadotropin-stimulated meiotic maturation and aging in vivo. *Biol. Reprod.* **43,** 870–6 (1990).
